# Family-centeredness of childhood obesity interventions: psychometrics & outcomes of the family-centered care assessment tool

**DOI:** 10.1186/s12955-020-01431-y

**Published:** 2020-06-11

**Authors:** Meg Simione, Mona Sharifi, Monica W. Gerber, Richard Marshall, Earlene Avalon, Lauren Fiechtner, Christine Horan, E. John Orav, Joseph Skelton, Elsie M. Taveras

**Affiliations:** 1 Division of General Academic Pediatrics, Department of Pediatrics, MassGeneral Hospital for Children, 125 Nashua Street, Suite 860, Boston, MA 02114 USA; 2 Department of Pediatrics, Harvard Medical School, Boston, MA USA; 3 Section of General Pediatrics, Department of Pediatrics, Yale University School of Medicine, New Haven, CT USA; 4 Department of Pediatrics, Harvard Vanguard Medical Associates, Boston, MA USA; 5 Northeastern University, Boston, MA USA; 6 Division of Pediatric Gastroenterology and Nutrition, MassGeneral Hospital for Children, Boston, MA 02114 USA; 7 Department of Medicine, Brigham and Women’s Hospital, Boston, MA USA; 8 Department of Pediatrics, Wake Forest School of Medicine, Winston-Salem, NC USA; 9 Department of Nutrition, Harvard T.H. Chan School of Public Health, Boston, MA USA

**Keywords:** Childhood obesity, Pediatric weight management, Family-centered outcomes, Primary care

## Abstract

**Background:**

Incorporating family-centered care principles into childhood obesity interventions is integral for improved clinical decision making, better follow-through, and more effective communication that leads to better outcomes and greater satisfaction with services. The purpose of this study is to evaluate the psychometric properties of a modified version of the Family Centered-Care Assessment (mFCCA) tool and to assess the family-centeredness of two clinical-community childhood obesity interventions.

**Methods:**

*Connect for Health* was a randomized trial testing the comparative effectiveness of two interventions that enrolled 721 children, ages 2–12 years, with a body mass index (BMI) ≥ 85th percentile. The two arms were (1) enhanced primary care; and (2) enhanced primary care *plus* contextually-tailored, health coaching. At the end of the one-year intervention, the mFCCA was administered. We used Rasch analyses to assess the tool’s psychometrics and examined differences between the groups using multiple linear regression.

**Results:**

629 parents completed the mFCCA resulting in an 87% response rate. The mean (SD) age of children was 8.0 (3.0) years. The exploratory factor analysis with 24 items all loaded onto a single factor. The Rasch modeling demonstrated good reliability as evidenced by the person separation reliability coefficient (0.99), and strong validity as evidenced by the range of item difficulty and overall model fit. The mean (SD, range) mFCCA score was 4.14 (0.85, 1–5). Compared to parents of children in the enhanced primary care arm, those whose children were in the enhanced primary care *plus* health coaching arm had higher mFCCA scores indicating greater perception of family-centeredness (β = 0.61 units [95% CI: 0.49, 0.73]).

**Conclusions:**

Using the mFCCA which demonstrated good psychometric properties for the assessment of family-centered care among parents of children with obesity, we found that individualized health coaching is a family-centered approach to pediatric weight management.

**Trial registration:**

Clinicaltrials.gov NCT02124460.

## Background

Family-centered care is a set of principles that aim to promote a partnership between families and health care practitioners through respect, trust, open and objective communication, and joint decision making [1–3]. The principles rely on underlying assumptions that parents know their children best, all families are unique, and supportive family and community contexts result in the best outcomes [4]. By incorporating family-centered care principles into pediatric care, it results in improved clinical decision making, increased follow-through, and more effective communication that leads to better child health outcomes and greater satisfaction with services [5, 6]. Given the high attrition rates of pediatric weight management programs, creating programs that focus on family-centered care by engaging families and addressing family’s concerns and priorities may improve satisfaction that may ultimately lead to less attrition in weight management programs [6, 7].

Having reliable and valid tools to measure the family-centeredness of clinical programs and research trials is important, as it will help to ensure these principles are incorporated into care. Examples of scales to assess family-centered care include the Medical Home Family Index [2] and the Consumer Assessment of Healthcare Providers and Systems Clinician and Group Survey [8]. Both instruments are widely used, but neither scale encompasses all the core principles represented in family-centered care. For example, they lack questions about honoring cultural diversity and traditions, and shared decision-making. The Family Centered Care Assessment (FCCA) is a tool for parents that was developed to evaluate the family-centeredness of healthcare services for children with special healthcare needs [9]. It was developed by experts from Family Voices, a family advocacy organization, the American Academy of Pediatrics, and the Maternal and Child Health Bureau. It was administered to 790 parents and was found to be reliable and valid [9]. A strength of the FCCA is the representation of eight family-centered care principles across the 24 questions which include communication with providers, decision-making interactions, future orientation and planning, strength-based care approach, care coordination, cultural and linguistic competent care, practice structure and policies that support family-centered care, and family support.

In the *Connect for Health* trial, a clinical-community childhood obesity intervention trial [10, 11], a modified version of the FCCA (mFCCA) that had more relevant items for parents of children with obesity than the original version was administered to parents. Our aims were to assess the psychometric properties of the mFCCA and to evaluate the family-centeredness of the *Connect for Health* trial for childhood obesity.

## Methods

We used data from the *Connect for Health* trial to assess the psychometric properties of the mFCCA and to evaluate differences in family-centeredness between the two intervention arms. *Connect for Health* was a blinded, randomized control trial testing the comparative effectiveness of two clinical-community childhood obesity interventions by examining child body mass index (BMI) z-scores and family-centered outcomes for childhood obesity [10, 11]. The trial was one-year and conducted in six pediatric practices in Massachusetts. A total of 721 children, ages 2–12 years, with a BMI ≥ 85th percentile for age and gender were enrolled in the trial. We randomized children into one of two intervention arms: [1] enhanced primary care (n = 361); or [2] enhanced primary care *plus* contextually tailored, health coaching (n = 360). The enhanced primary care arm included electronic health record enhancements such as best practice alerts, clinical decision support tools, educational materials; and families received neighborhood resource guides and monthly text messaging. The health coaching arm received the electronic health record enhancements in addition to contextually-tailored health coaching support via six telephone/ video contacts, an online, interactive community resource map, and twice-weekly text messaging. Both arms were designed to be family-centered, for example, through educational materials and connecting families to resources. The study design, setting, details of the intervention, and results of the primary outcomes of the trial have previously been described in more detail [10, 11]. The Partners Institutional Review Board approved the trial and it was registered in clinicaltrials.gov.

### Development of the mFCCA

At the end of the one-year intervention, we administered the mFCCA to parents. The mFCCA was developed with the input of the authors of the FCCA [9]. Five questions from the original scale were replaced with questions that were more applicable to children with obesity. The original authors provided alternate questions from their previously developed question bank that preserved the psychometrics of the scale. We included questions about addressing parental concerns, helping to find resources, identifying support systems, promoting mental health, and planning for changes in weight management or behavior change routines. These questions replaced items regarding disagreeing with healthcare professionals about recommendations, modifying daily care and treatment routines, ways to pay for things that insurance does not cover, understanding content in the medical record, and discussing hopes for the child’s future. Study investigators reviewed the final questions to ensure content validity for childhood obesity. The modified scale had 24 items that represented principles of family-centered care that resulted in eight different topical areas similar to the original scale. Responses to the scale were ordinal ranging from 1 to 5 with higher scores representing a greater perception of family-centeredness. A “not applicable” response was also provided.

### Psychometric analyses of the mFCCA

Our first objective was to assess the psychometric properties of the mFCCA using Rasch modeling [12–15]. The original scale was developed using item response theory, and those analyses were replicated in this study. Only children in the enhanced primary care arm were included in the psychometric analyses as this arm answered questions in relation to services from primary care providers (rather than health coaches) which is more representative of usual care. Because we made modifications to items, we performed an exploratory factor analysis using the principal axis method for factor extraction to confirm the uni-dimensionality of the scale rather than performing a confirmatory factor analysis. Prior to analysis, we determined that any item with a low factor loading < 0.4 would be deleted as was done in the original analyses [9]. We also examined the Scree plot and reviewed the tests of the hypothesis results. We calculated item total correlations to examine the homogeneity of the scale and determined any correlations < 0.3 would be deleted. We then used Rasch analysis using a partial credit model to assess the overall fit of the items [16–18]. We calculated Chi-square based itemfit statistics that explain how well the data fit the model, including infit and outfit statistics that detected inliers and outliers, respectively. We set our criteria for item inclusion to be within the range of 0.5–1.5 [19, 20]. For each item, we also calculated standard error of the item score and item difficulty. Item difficulty was expressed using a logit scale (centered at 0) that ranges from negative (represents easy items) to positive (represents more difficult items) and measures how challenging it is to implement each item into clinical practice [13]. “Easy” items represent principles of family-centered care that would be easy to incorporate into care (i.e., taking time to address family’s concerns), whereas “difficult” items would be more challenging to incorporate into clinical practice. To test for potential question bias, we performed Differential Item Functioning (DIF) [21, 22], a statistical test that examines whether an item is measuring different abilities for subgroups, and tested for sex, income, race, and ethnicity. For the above analyses, “not applicable” responses were set to missing because they were considered structurally missing and mean imputation was used for other missing responses [23]. We deleted items that did not fall within acceptable predetermined ranges of the described analyses and the Rasch analysis was iterated until all items demonstrated a good fit. Once a final set of items was determined, we calculated person separation reliability to assess the internal consistency of the scale. This metric is equivalent to a Cronbach’s alpha [24, 25].

### Family-Centered Outcomes & Statistical Analyses

Our second aim was to determine the family-centeredness of the two intervention arms. First, we described overall participant characteristics according to intervention arm. For each participant, we derived a score by calculating the mean of the final items (items that were determined to show good model fit). Due to missing data, we calculated mean scores rather than a total score as was done in the original paper. We treated “not applicable” and missing responses in a similar manner as we did for the psychometric analyses described above. We used multiple linear regression adjusting for study site to examine the outcomes between the two intervention arms and determine the regression co-efficient and 95% confidence intervals. We set our alpha level at 0.05 to test for statistical significance. All analyses were performed using R version 3.4.4 and the eRM and lordif packages [16, 22, 26].

## Results

After the one-year intervention, 629 parents completed the mFCCA resulting in an 87% response rate. Participants who had “not applicable” or missing responses for > 50% of items were excluded from the analyses. A total of 316 children in the enhanced primary care arm and 313 children in the enhanced primary care *plus* health coaching arm were included in the final analyses. Overall, the mean (SD) age of children enrolled in the study was 8.0 (3.0) years, and 35.5% of children were non-Hispanic White, 34.2% were non-Hispanic Black, 21% were Hispanic, and 9.4% were other races. Table 1 shows characteristics of the children and their parents.

**Table 1:** Child, Parent, and Household Characteristics According to *Connect for Health* Intervention Arm

	No. (%)			
	Overall	Enhanced Primary Care	Enhanced Primary Care plus Health Coach	***P*** Value
	N = 629	N = 316	N = 313	
**Child Characteristics**				
Age, mean (SD)	8.04 (2.98)	7.96 (3.0)	8.12 (2.96)	0.52
Sex				0.55
Male	301 (47.90%)	147 (46.5%)	154 (49.2%)	
Female	328 (52.1%)	169 (53.5%)	159 (50.8%)	
Race/ ethnicity				0.32
Non-Hispanic white	223 (35.5%)	119 (37.7%)	104 (33.2%)	
Non-Hispanic black	215 (34.2%)	99 (31.3%)	116 (37.1%)	
Hispanic/Latino	132 (21.0%)	71 (22.5%)	61 (19.5%)	
Other	59 (9.38%)	27 (8.54%)	32 (10.2%)	
BMI, mean (SD)	23 (4.81)	23 (4.62)	23 (5.01)	0.82
BMI z-score, mean (SD)	1.89 (0.52)	1.91 (0.51)	1.87 (0.53)	0.28
**Parent Characteristics**				
Age, mean (SD)	38.6 (7.31)	38.8 (7.57)	38.3 (7.04)	0.37
BMI				
< 25	140 (22.8%)	70 (22.9%)	70 (22.7%)	0.99
25–29	210 (34.1%)	105 (33.2%)	105 (34.0%)	
≥ 30	265 (43.1%)	131 (41.5%)	134 (43.4%)	
Income				0.03
≤ $50,000	261 (42.3%)	118 (37.9%)	143 (46.7%)	
> $50,000	356 (57.7%)	193 (62.1%)	163 (53.3%)	
Education, < college graduate	302 (48.2%)	163 (51.6%)	139 (44.7%)	0.10

### Psychometric analyses

**Fig. 1:**
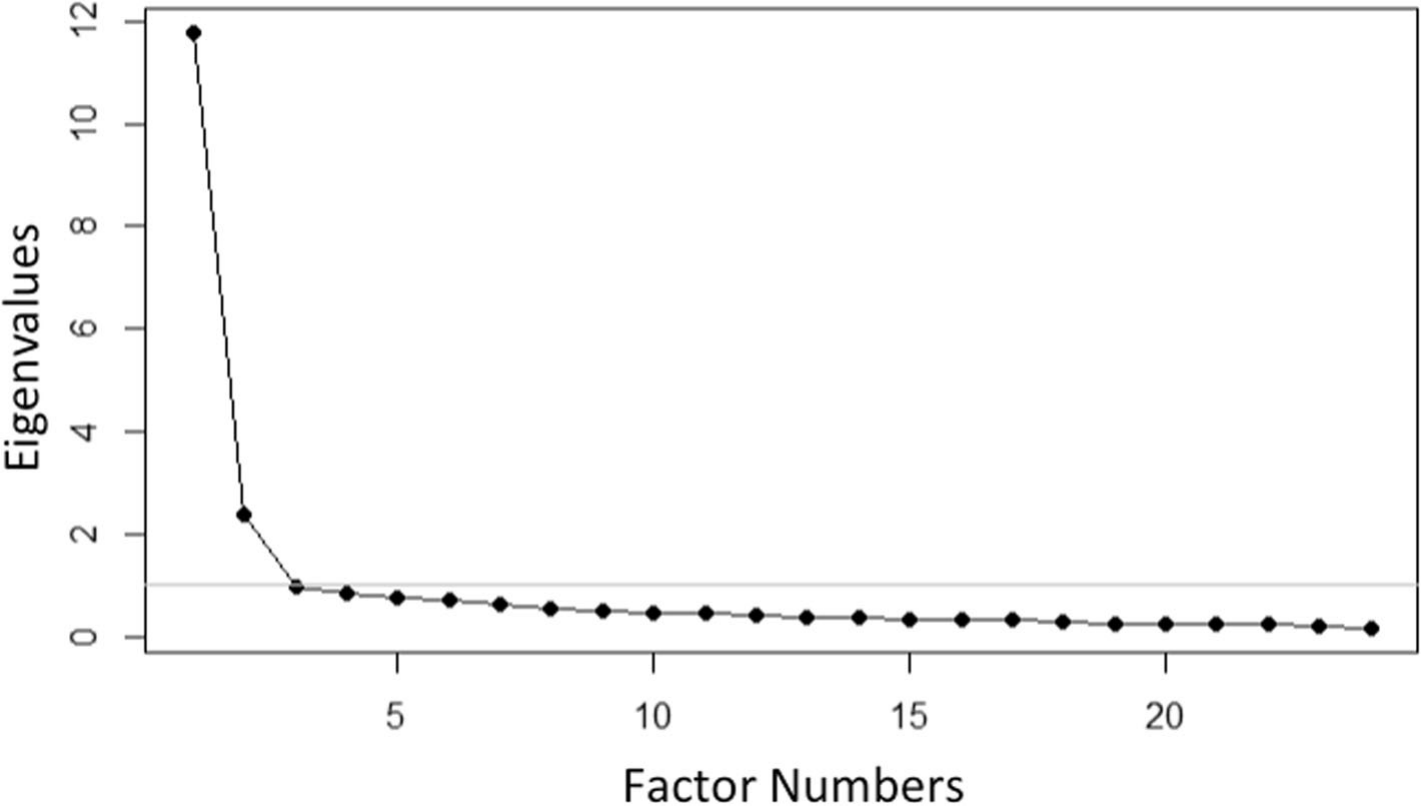
Exploratory factor analysis Scree plot of the modified Family-Centered Care Assessment Tool

For the psychometric analysis, 316 responses (enhanced primary care arm only) were included. The results of the exploratory factor analysis revealed the presence of a single factor (eigen value = 11.24) which explained 47% of the variance (see Fig. 1). Individual item factor loadings were all > 0.4 and the item total correlations for the individual items were all > 0.3. Based on the factor loadings and item total correlations, no items were deleted at this stage of the analyses. Rasch item fit statistics revealed that 22 items fell between the range of 0.5–1.5. The two items (items #8 and #13) that were outside of the range were not deleted from the final scale because of their strong factor loadings (0.82 and 0.83, respectively) and strong item total correlations (0.75 and 0.76, respectively). The questions showed a broad range of item difficulty ranging from − 1.22 logits (easiest) to 1.10 logits (most difficult). The DIF analyses did not reveal question bias for sex, income, race, and ethnicity as the pseudo R^2^ measures were < 0.02. In a no DIF condition, we would expect that pseudo R^2^ measures to be < 0.02 [22]. Results of the psychometric analyses are shown in Table 2. Based on the psychometric analyses, we did not remove any items and the final 24 items represented eight core principles of the family-centered care. After finalizing the items of the mFCCA, we calculated the person separation reliability which was 0.99 revealing high internal consistency of the scale.

**Table 2:** Estimates of Item Difficulty, Standard Error, Mean-Square Fit Statistics, Item–Total Correlations, and Topical Area of the Modified Family Centered Care Assessment Tool

Item #	My child’s health care provider/ health coach …	Item Difficulty	Standard Error of Item Score	Infit Mean-Square Value	Outfit Mean-Square Value	Item Total Correlation	Topical Area
4^a^	Takes enough time to address my concerns	−1.22	0.03	0.84	0.96	0.59	Communication
7	Talks with me about my child’s overall health and well-being	−1.09	0.03	0.82	0.58	0.63	Future Promotion
1	Talks with me using words I understand	−0.93	0.03	1.26	1.16	0.51	Communication
2	Supports me in the role that I want to take	−0.89	0.03	0.95	0.74	0.59	Decision Making
12	Recognizes my strengths in caring for my child	−0.76	0.04	0.99	0.90	0.61	Strength-Based
10	Considers my schedule before making appointments or phone calls	−0.62	0.04	1.35	1.42	0.51	Practice Structure
8^a^	Has a way to help find information and resources	−0.51	0.04	0.65	0.42	0.75	Future Promotion
3	Decides together on goals	−0.44	0.05	0.88	0.81	0.69	Decision Making
13	Works with me to adjust our plan	−0.36	0.05	0.69	0.47	0.76	Family Support
6	Works with me to plan when there are big changes	−0.33	0.05	0.89	1.16	0.71	Future Promotion
11	Asks me what is working well	−0.24	0.04	0.70	0.87	0.74	Strength-Based
23^a^	Talks with me about my child’s social and emotional wellbeing	−0.22	0.04	1.07	1.05	0.6	Future Promotion
9	Offers ways to provide support where my child usually spends time	−0.15	0.05	0.85	0.67	0.73	Practice Structure
5	Talks with me about how decisions will affect my family	−0.07	0.05	0.88	0.76	0.72	Decision Making
15	Asks about issues that affect the well-being of my family	−0.04	0.05	1.02	0.77	0.69	Family Support
14	Asks me about health or emotional stresses I have	0.10	0.06	1.02	0.82	0.69	Family Support
24^a^	Helps me plan when changes in my child’s routine are needed	0.14	0.04	0.85	0.87	0.71	Family Support
19	Has a way to help me make contact with community resources	0.23	0.06	0.93	0.81	0.73	Care Coordination
22^a^	Asks me about where I turn to for support	0.54	0.05	0.97	0.95	0.68	Family Support
17	Asks about my family’s beliefs and practices	0.70	0.07	1.11	1.20	0.66	Cultural Competence
21	Gives me information to help other people understand	0.85	0.07	1.10	1.01	0.67	Family Support
18	Asks if we want to integrate alternative practices into plans	0.89	0.07	1.05	0.97	0.66	Cultural Competence
16	Asks if I would like other community members to help make decisions	1.05	0.07	1.16	1.16	0.63	Cultural Competence
20	Has a way to connect me with other families	1.10	0.08	1.36	1.32	0.59	Family Support

Items with ^a^ represent questions that were modified from the original scale

### Family-centered outcomes

**Fig. 2:**
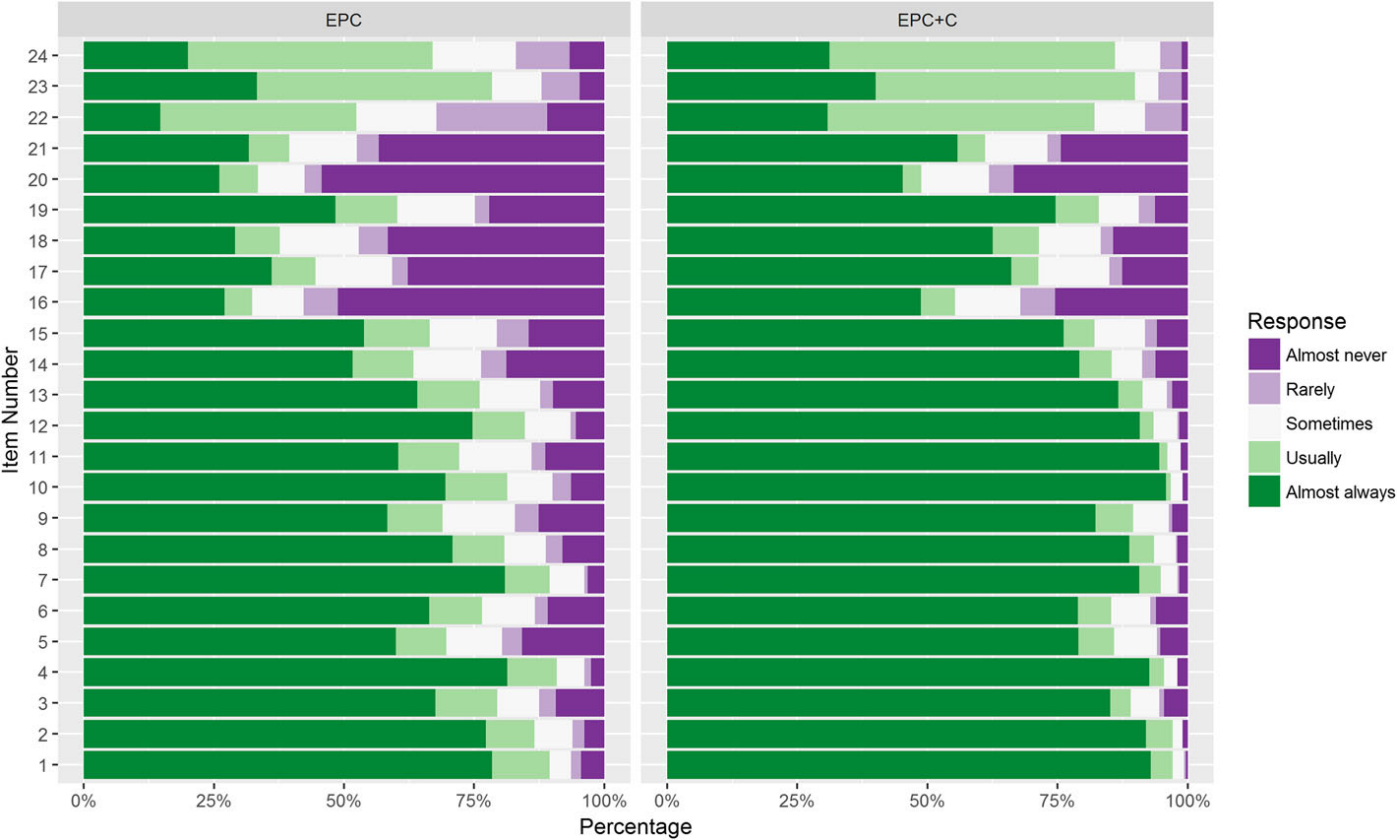
The mean responses for the final items in the modified Family-Centered Care Assessment Tool for both intervention arms. EPC = Enhanced primary care. EPC + C = Enhanced primary care *plus* health coaching

The mean score (SD) for the final 24 items of the mFCCA was 3.84 (0.95) for the enhanced primary care arm and 4.45 (0.61) for the enhanced primary care *plus* health coaching arm (Table 3). Figure 2 shows the mean responses for the final items on the mFCCA for both intervention arms. Compared to parents of children in the enhanced primary care arm, those whose children were in the enhanced primary care *plus* health coaching arm had higher mFCCA scores indicating greater perception of family-centeredness (β = 0.61 units [95% CI: 0.49, 0.73]).

**Table 3:** Difference in Family-Centered Care Outcomes Across the Two Intervention Arms of *Connect for Health*

	Mean (SD)	β Value (95% CI)	
Study Arm	1-y Follow-up	Difference	*p*-value
**FCCA mean score**			
Enhanced primary care plus health coach	4.4 (0.87)	0.61 (0.49, 0.73)	<.0001
Enhanced primary care	3.8 (0.87)	0.0 [Reference]	

Possible range of FCCA mean score: 1 (almost never) to 5 (almost always)

## Discussion

The purpose of this study was to evaluate the psychometric properties of the mFCCA and to assess the family-centeredness of the *Connect for Health* trial for childhood obesity. We found the mFCCA to be a reliable and valid instrument for assessing family-centeredness. We also found that the intervention arm that received enhanced primary care *plus* health coaching had a higher score on mFCCA indicating a greater perception of family-centeredness than the intervention arm that received enhanced primary care only.

Using exploratory factor analysis, we determined that the 24 items in the mFCCA all loaded onto a single factor; and using Rasch analysis we determined the scale had good reliability as evidenced by the high person separation reliability coefficient, and strong validity as evidenced by the range of item difficulty, the absence of DIF items, and the overall model fit. Our findings were similar to the original version of the FCCA [9] despite exchanging five questions from the original version and testing the scale on a different population (children with overweight or obesity v. children with special healthcare needs). The mFCCA had a range of item difficulties, which is important in item response theory to distinguish performance between high and low performers. For example, if a scale only had “easy” questions then we would be unable to discern programs that had a high degree of family-centeredness from a low degree. Interestingly, several of the items that were found to be the most difficult and less likely to be incorporated into care, were items representing cultural and linguistic competence in care which is a principle not represented in other family-centered scales [2, 8, 9]. Given the widening gap of racial/ ethnic disparities in childhood obesity rates [27], these principles represent important components of family-centered care that ensures all families are engaged in services.

After assessing the reliability and validity of the scale, we then evaluated the family-centeredness of the interventions. The intervention arm that received enhanced primary care *plus* health coaching was found to have a greater perception of family-centered care than the arm that received enhanced primary care only. The health coaching arm had a mean score of 4.5 indicating that most responses fell between “usually” or “almost always”, whereas the enhanced primary care arm had a mean score of 3.8 indicating that most responses fell between “sometimes” or “usually”. The parents in the enhanced primary care *plus* health coaching arm tended to score items that were more difficult (based on item difficulty scores) higher than the other arm did suggesting that the services they received incorporated in more family-centered care principles. Other notable item-level differences between the two arms included using language that a family understands, considering the family’s schedule when making appointments, and asking about what is working. Over 90% of respondents in the enhanced primary care *plus* health coaching arm answered “almost always” as compared to less than 70% in the enhanced primary care arm. The enhanced primary care *plus* health coaching arm may have perceived their care as more family-centered due to numerous aspects of the design of the intervention, including regular contact with a health coach through phone-calls or video chats, interactive text messaging with behavioral goals [28], and the tailored family resources provided to them. As previously reported, this arm was also found to have better adherence to the different components of the intervention including the text messaging program and use of the neighborhood resource guide [11]. Although the enhanced primary care *plus* health coaching arm received a higher dosage of the intervention, based on the individual item level differences between the two arms, it appears that the differences in family-centeredness may be attributable to other factors besides amount of care and attention. For example, the enhanced primary care *plus* health coaching arm were more likely to respond “almost always” to questions relating to using understandable words, asking what is going well, connecting family to resources, and discussing the child’s overall health and well-being.

Most childhood obesity interventions are not family-centered [29] and that may help to explain the high attrition rates of weight management programs [30] and poor outcomes. For programs to help with lifestyle modifications [31] and behavioral changes, providers must be able to target, for example, goal setting, monitoring, and problem-solving while applying the principles of family-centered care [32]. In a review of childhood obesity interventions, Gallo and colleagues [33] found that the more family-centered an intervention was, the better the health outcomes of the children. This finding reinforces the importance of family-centered care in weight management programs. In the *Connect for Health* trial, as previously reported [11], children in both intervention arms showed improvements in their BMI z-scores. The higher perception of family-centered care in the enhanced primary *plus* health coaching arm may, though, help to explain the improved child health-related quality of life and it is possible that these children may show long-term improvements that persist after the intervention ends. While weight and physical health are primary objectives of pediatric weight management, outcomes such as quality of life and overall wellbeing are important as well [34].

Our study had several limitations. Family-centered care was a secondary outcome of this trial and was assessed after the intervention was completed, therefore we cannot draw conclusions about family-centeredness pre/post intervention. We calculated the mean of the responses on the mFCCA, whereas the original scale used a total score, therefore we were not able to use the cut-off scores that were applied in the original scale. The original scale calculated a neutral score and a score that indicated a high perception of family-centeredness. A cut-off score would be beneficial for assessing the quality of weight management programs. In addition, the scale contained 24 items that can be time consuming to administer which increases the likelihood of parents not completing the scale or skipping individual items. For this study our response rate was high, but if providers were administering this in busy waiting rooms the length of the scale may affect compliance. Because the scale loaded onto one factor, it provides opportunities in the future to develop a shortened scale that retains the psychometrics and the family-centered care principles.

## Conclusion

In conclusion, the mFCCA for childhood obesity was found to be a reliable and valid instrument for assessing the family-centeredness of pediatric weight management programs. The mFCCA retained key principles for family-centered care and to our knowledge is the only existing scale to assess family centeredness of childhood obesity programs. By developing this tool to measure the family-centeredness of childhood obesity services, we are now able to evaluate the family-centeredness of programs and ensure optimal care is being delivered to children and their families. Our findings also suggest that pediatric weight management programs that include contextually-tailored, individual health coaching are perceived to be more family-centered in comparison to enhanced primary care alone. Pediatric weight management programs should assess the extent to which their services are family-centered and consider incorporating the mFCCA measure in their evaluation. Future studies should also examine the extent to which greater family-centeredness is associated with long term adherence, attrition, and weight status outcomes.

## Data Availability

The datasets used during the current study are available from the corresponding author on reasonable request.

## Abbreviations

FCCA: Family Centered Care Assessment tool
mFCCA: Modified version of the Family Centered-Care Assessment tool;
BMI: Body mass index
DIF: Differential Item Functioning
